# An edge-aware salient context fusion and refinement network for hippocampal segmentation in MR images and its diagnostic value for mild cognitive impairment

**DOI:** 10.3389/fneur.2026.1899123

**Published:** 2026-07-17

**Authors:** Limin Liu, Xiaolong Chen, Qiqun Zeng, Shili Zhou, Xia Zhang

**Affiliations:** 1 Department of Ultrasound Medicine, The Second Affiliated Hospital, Hengyang Medical School, University of South China, Hengyang, China; 2 Department of Radiology, The First Affiliated Hospital, Hengyang Medical School, University of South China, Hengyang, China

**Keywords:** deep learning, ESCFR-net, hippocampus volume, mild cognitive impairment, MRI

## Abstract

**Background:**

Accurate assessment of hippocampal volume is of significant clinical value for the early diagnosis and disease monitoring of Alzheimer’s disease (AD). However, automatic segmentation of the hippocampus in MR images remains challenging due to its elongated and irregular morphology, blurred boundaries, low contrast with surrounding tissues, and substantial inter-individual anatomical variability.

**Methods:**

We propose an Edge-aware Salient Context Fusion Refinement Network (ESCFR-Net). Built upon a classic U-shaped encoder-decoder architecture, the proposed network employs a Salient Feature Enhancer to suppress background interference and enhance weak feature responses of the hippocampus. A Global Channel Context Attention (GCCA) module is introduced to model long-range spatial dependencies, while a Multi-scale Context Fusion Refinement Module (MCFRM) improves the utilization of multi-scale features. Furthermore, an Edge-Guided Refinement Attention (EGRA) module synergistically enhances edge and semantic features to precisely delineate weak boundaries.

**Results:**

Experimental results on a self-constructed dataset comprising 225 3D-T1 MRI scans demonstrate that ESCFR-Net achieves a Dice coefficient of 0.9004, outperforming state-of-the-art methods such as SwinUNETR and PMFS-Net. Clinical association analysis, conducted on 91 healthy controls (HCs) and 91 patients with mild cognitive impairment (MCI), reveals that bilateral hippocampal volumes in MCI group are significantly smaller than those in HCs (*p* < 0.001). Additionally, the total hippocampal volume achieves an area under the curve (AUC) of 0.927 in distinguishing HCs from patients with MCI, with sensitivity and specificity reaching 90.11 and 83.52%, respectively.

**Conclusion:**

This study provides a highly accurate and robust automated hippocampal segmentation tool for early diagnosis, disease monitoring, and clinical decision-making in Alzheimer’s disease.

## 1 Introduction

Alzheimer’s disease (AD) is a neurodegenerative disease clinically characterized by progressive decline of cognitive function, remains a leading cause of dementia worldwide, with rising prevalence posing an escalating global health burden (1). In most cases, AD initially presents with isolated memory loss, a condition classified as mild cognitive impairment (MCI) and eventually evolves to overt dementia. Early intervention, which can slow disease progression, hinges on the identification of reliable biomarkers. Several studies found that lower hippocampal volume in the early MCI phase is related to progression to AD dementia, which had become a biomarker suggestive of neurodegeneration in the diagnostic criteria for MCI caused by AD (2–4). Hence, a robust and reliable method for assessing hippocampal volume is essential for early diagnosis, disease tracking, and treatment planning in clinical settings of AD.

Volume assessments of structures of interest can be achieved through segmentation of magnetic resonance (MR) images, which has been commonly applied to measure the hippocampus (5–7). Hippocampal segmentation can be performed either manually or automatically. Manual segmentation is the gold standard in clinical practice, while it is time-consuming, rater-dependent, and heavily influenced by the rater’s experience (8, 9). Indeed, it is this labor-intensive and operator-dependent nature of manual segmentation that hinders its clinical application. As a result, although associations between hippocampal volume changes and cognitive decline have been observed, systematic evaluation of this volume-cognition relationship remains limited. Thus, there is a growing need for automated, protocol-independent hippocampal segmentation methods to enable robust, large-scale evaluation of volume-cognition relationships in AD.

Deep learning has been widely applied in many fields, such as classification and segmentation (10–12). However, the hippocampus is relatively small and irregularly shaped, lacks clear boundaries, and varies considerably across individuals in MR images, posing significant challenges for the task of hippocampal automatic segmentation (13). With the development of deep learning, medical image segmentation has achieved significantly improved in recent years. Fully Convolutional Networks (FCN) make classification of image-level further extended to pixel-wise, becoming a milestone in the field of semantic segmentation (14). However, the final segmentation size of FCN is smaller than the original image, and the result is relatively rough. Subsequently, The encoder-decoder architecture, represented by U-Net, has become a mainstream foundational framework by virtue of its skip connections and end-to-end optimization (15).

Early hippocampal segmentation methods based on convolutional architectures predominantly used U-Net and its variants as the base framework, leveraging multi-scale convolutions, residual connections, and attention mechanisms to strengthen local detail extraction and hierarchical feature representation (16–18). Among these, Light3DHS integrates multi-scale convolutions with Transformer-based modeling approaches, accommodating the learning of the hippocampus’s elongated and irregular anatomical shape (19). PMFSNet employs a polarized multi-scale self-attention network to enhance cross-level feature correlations, thereby adapting to inter-individual morphological variations of the hippocampus (20). MWFNet integrates wavelet transform with a multi-scale attention residual structure to compensate for the loss of fine-grained details in small hippocampal regions during convolutional downsampling (21). FED-UNet++ and DPNU-Net, through nested decoding and progressive feature aggregation, respectively, further improve the reconstruction accuracy of weak hippocampal boundaries (22, 23). Models such as SwinUNETR and UNETR incorporate Transformers into the encoding pathway, significantly enhancing global long-range dependency modeling and multi-scale contextual representation (24, 25). Although these convolutional methods and their variants excel at local texture and fine-grained feature extraction, they still suffer from inherent limitations, including weak global topology perception, a high risk of missing small hippocampal structures, and difficulty in accurately distinguishing weak boundaries from adjacent adhesive tissues.

To overcome the limitation of insufficient long-range modeling capability in pure convolutional networks, hybrid architectures integrating CNNs and Transformers have gradually become a mainstream research direction for hippocampal segmentation. These architectures can simultaneously capture local detail representation and global semantic correlation learning (26). TransBTS and CoTr adopt a serial fusion paradigm of convolutions and Transformers to enhance the perception of global spatial topological structures of the hippocampus (27, 28). PHTrans employs a dual-branch parallel architecture to fuse the local representation advantages of convolutions with the global modeling characteristics of attention mechanisms, thereby improving the segmentation robustness of the hippocampus with complex morphology (29). DSnet and spatial depth-attention U-Net leverage a dual-branch structure coupled with cross-dimensional attention mechanisms to specifically enhance feature representation of the hippocampal tail boundary under low-contrast conditions (30, 31). Although existing hybrid models have achieved improvements in global context capture, they generally suffer from high feature redundancy and structural complexity. Their adaptability to small hippocampal targets and blurred boundaries remains insufficient, making it difficult to simultaneously satisfy the dual requirements of high-precision clinical segmentation and practical applicability.

Although existing segmentation methods have made certain progress in global context modeling, edge contour optimization, and multi-scale feature fusion, three major challenges remain for automated segmentation of the hippocampus in the brain. First, the hippocampus is a small, slender target structure in the brain, characterized by substantial inter-individual morphological variations and uneven scale distribution. Its fine-grained regional features are easily overwhelmed by surrounding brain tissue backgrounds, making effective alignment and deep fusion of multi-scale features difficult. In addition, the hippocampus exhibits highly similar intensity profiles to adjacent gray matter, white matter, and ventricular tissues, with extremely blurred boundaries and frequent adhesion to neighboring brain structures. Conventional attention and convolution mechanisms struggle to accurately distinguish such weak edge contours. Moreover, due to its elongated, strip-like irregular morphology, the hippocampus involves complex long-range spatial dependencies. Standard networks are often incapable of effectively modeling global morphological topological information, leading to issues such as regional missing segmentation, adhesive misclassification, and contour discontinuities. Effectively addressing challenges of feature submergence of small targets, difficulty in distinguishing blurred boundaries, and insufficient global topological modeling has become a critical step toward advancing intelligent hippocampal segmentation toward clinical application.

On the basis of previous researches, this paper targets high-precision automatic segmentation of the hippocampus. We propose the Edge-aware Salient Context Fusion Refinement Network (ESCFR-Net), which builds upon the classic U-shaped encoder-decoder architecture. The network integrates four core modules: Salient Feature Enhancer (SFE), Global Channel Context Attention (GCCA), Multi-scale Context Fusion Refinement Module (MCFRM), and Edge-Guided Refinement Attention (EGRA). The SFE module enhances feature responses of small hippocampal targets while suppressing complex background interference. The GCCA module introduces Global Channel Context Attention to capture long-range global morphological dependencies of the elongated hippocampus, strengthening global semantic guidance. The MCFRM performs cross-level feature alignment, contextual attention coupling, and feature refinement to accommodate inter-individual morphological variations. The EGRA module enhances weak boundary representation, separates adherent adjacent tissues, and precisely delineates fine contours. To address challenges including morphological variability, blurred boundaries, and small-target omission, a tailored loss function can be incorporated to further improve segmentation stability and generalization. Experimental results show that ESCFR-Net outperforms existing state-of-the-art methods in segmentation accuracy, weak boundary continuity, small-target detection, and background interference resistance. The method demonstrates robust performance across hippocampi with varying morphologies and boundary conditions, showing strong potential for clinical application in brain MRI hippocampal segmentation.

The main contributions of this work are summarized as follows:

1. We propose an Edge-aware Salient Context Fusion Refinement Network (ESCFR-Net) for hippocampal segmentation. It organically combined saliency enhancement, global context modeling, multi-scale fusion, and edge refinement into a complete inference pipeline of feature enhancement, global modeling, cross-layer fusion, and edge calibration. By synergistically adapting to hippocampal anatomy, ESCFR-Net achieves high-precision and high-robustness automatic segmentation.
2. We establish a collaborative saliency-global context mechanism, where SFE enhances small-target features and suppresses background interference, and GCCA models long-range topological dependencies of the elongated hippocampus. Together, these modules enhance feature representation from both local and global perspectives, reducing missing segmentation and contour discontinuities.
3. We propose a joint optimization strategy of multi-scale feature fusion and edge awareness is established. MCFRM combined EGRA to achieve coarse feature refinement and weak-boundary restoration, effectively resolving misclassifications due to blurred boundaries and tissue adhesion.

## 2 Methods

The study protocol was conducted in accordance with the Helsinki Declaration of 1975 and approved by the local ethics committees. Written informed consent was waived due to the retrospective nature of the study.

### 2.1 Study design

In this study, we collected 225 three-dimensional T1-weighted (3D-T1) sequence MRI scans to construct a dataset for the development and evaluation of the automatic hippocampal segmentation method. This image dataset was acquired from 225 patients who underwent 3D-T1 sequence MRI scanning from June 2023 to May 2025 at our hospital. We divide 225 data into training set, validation set and test set according to 8:1:1, with images for 180, 23 and 22 patients, respectively. The images for the first group of 180 patients were used to train the hippocampus segmentation model. The images for the second group of 23 cases were used to fine-tune the hyperparameters of the model. The images for the third group of 22 cases were used as the test set to evaluate the performance of the segmentation model.

Among these 225 patients, 43 were excluded from the evaluation of the correlation between hippocampal volume and cognitive impairment. The exclusion criteria were as follows: (1) patients who could not complete the Mini-Mental State Examination (MMSE); (2) patients with a history of mental and/or neurological diseases; (3) those with organic diseases of the nervous system; (4) a history of alcohol, smoking, or drug abuse; (5) patients with contraindications for MRI. The MMSE is a 30-point questionnaire used extensively in clinical and research settings to measure cognitive impairment. A diagnosis of MCI was established using education specific cutoff points of total scores of MMSE. MMSE scores less than 19 for illiterate individuals, 22 for participants with elementary school education, and 26 for those with middle school education and above (32). The final 182 participants were divided into two groups: the healthy control (HC) group (*n* = 91), participants with MCI (*n* = 91).

For model training, all original 3D volumetric MRI scans were decomposed into consecutive 2D axial slices. All slices were resized to 512 × 512. The input tensor adopts the format 8 × 1 × 512 × 512, corresponding to a batch size of 8. This 2D slice-based input strategy matches the encoder–decoder architecture and ensures stable training.

### 2.2 Automatic hippocampus segmentation model

**Figure 1:**
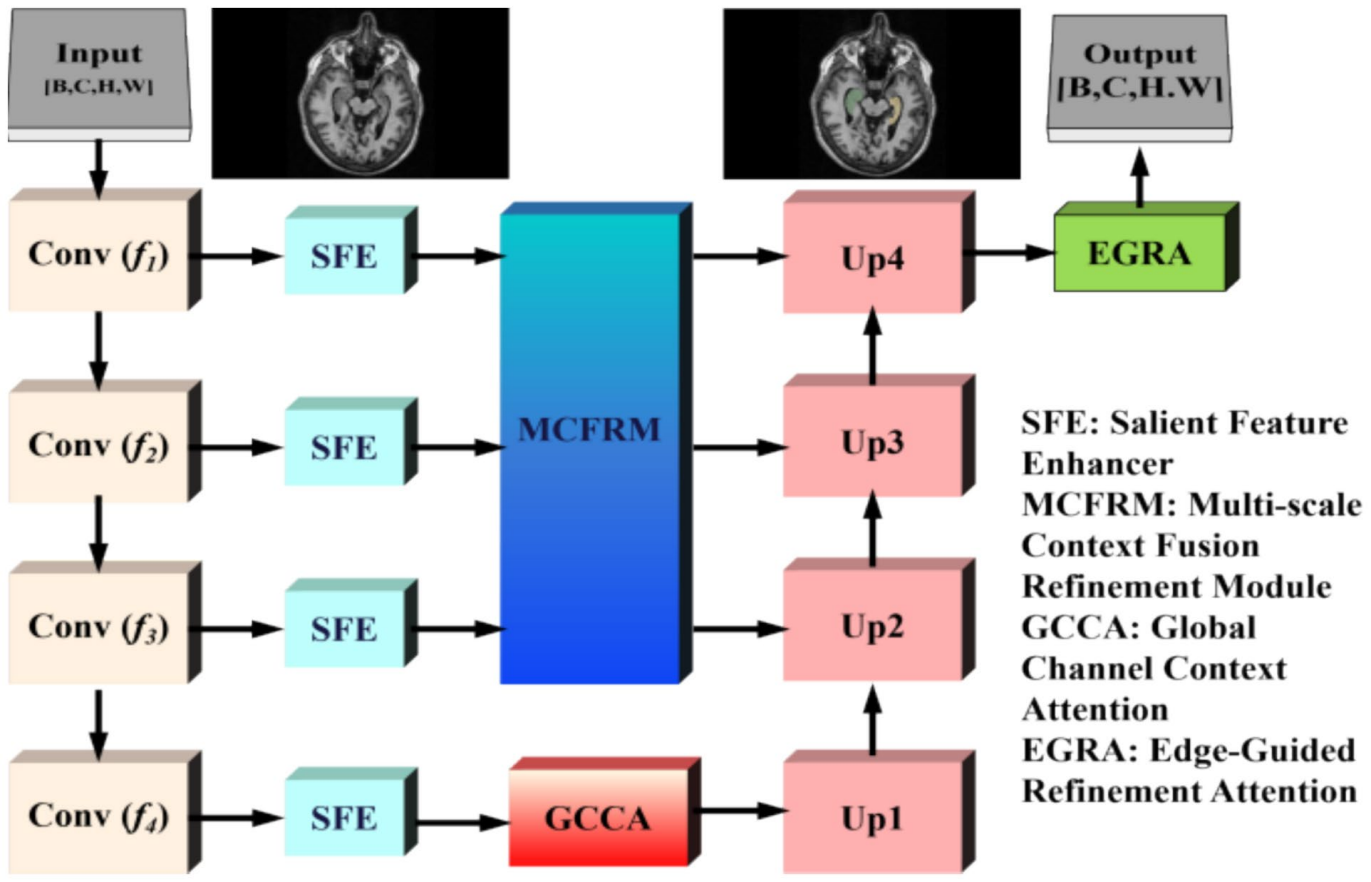
Edge-aware Salient Context Fusion Refinement Network (ESCFR-Net).

To address the challenges of multi-scale target adaptation, blurred boundaries, and insufficient global context modeling in automatic hippocampal segmentation, this paper proposes the Edge-aware Salient Context Fusion Refinement Network (ESCFR-Net) for high-precision and robust hippocampal segmentation. As shown in Figure 1, the network employs a U-shaped encoder-decoder framework integrating four core modules: Salient Feature Enhancer (SFE), Global Channel Context Attention (GCCA), Multi-scale Context Fusion Refinement Module (MCFRM), and Edge-Guided Refinement Attention (EGRA). This integration establishes an end-to-end segmentation pipeline featuring multi-scale coordination, multi-attention synergy, and bidirectional boundary-semantic enhancement, effectively resolving key challenges such as large scale variations, blurred and adhesive weak boundaries, strong background interference, and global context modeling difficulties in medical image segmentation.

#### 2.2.1 Illustration of whole network structure

The encoder applies four down-sampling operations to the input brain MRI image (uniformly resized to 512 × 512 with single channel), gradually building a multi-scale feature pyramid from low-level detailed features ( $f_{1}$ ) to high-level semantic features ( $f_{4}$ ). By hierarchically modeling the image with different receptive fields, the network thoroughly captures the structural and contextual information of the hippocampus, laying a multi-dimensional foundation for subsequent fine-grained modeling. All feature levels ( $f_{1}$ to $f_{4}$ ) with explicit hierarchical dimensions of *f*_1_ (64 channels, 512 × 512), *f*_2_ (128 channels, 256 × 256), *f*_3_ (256 channels, 128 × 128) and *f*_4_ (512 channels, 64 × 64) are then processed by the SFE module, which performs joint channel-spatial enhancement to produce calibrated enhanced features ( $f_{1}^{s},f_{2}^{s},f_{3}^{s},f_{4}^{s}$ ). This design not only suppresses complex cerebral background noise but also adaptively boosts the feature response of the small-target hippocampus, thereby significantly improving the model’s recognition capability and feature discriminability for weak signals.

To model the global spatial dependencies of high-level semantic features, the highest-level enhanced feature $f_{4}^{s}$ is fed into the Global Channel Context Attention (GCCA). This module employs a lightweight multi-head cross-dimensional attention mechanism to efficiently model the global morphological associations of the elongated and irregular hippocampal structure under low computational complexity constraints, while simultaneously suppressing background noise interference. It outputs an optimized high-level semantic feature $f_{4}^{\text{gcca}}$ , which provides strongly discriminative semantic guidance for the decoder, thereby enhancing the model’s ability to perceive and adapt to inter-individual morphological variations of the hippocampus.

The first three levels of enhanced features $f_{1}^{s},f_{2}^{s},f_{3}^{s}$ are then input into the Multi-scale Context Fusion Refinement Module (MCFRM), which performs dimensional alignment, spatial registration, and contextual attention coupling refinement across scales. This generates feature representations that strictly match the decoder hierarchy, effectively alleviating the problems of insufficient multi-scale feature utilization and semantic discontinuity between levels, and significantly improving the quality of skip connection feature transmission.

The decoder employs four stages of up-sampling (Up1–Up4) along with skip connection mechanisms to progressively fuse the refined features from MCFRM, the corresponding SFE-enhanced features at each scale, and the high-level guidance feature $f_{4}^{\text{gcca}}$ , achieving coarse-to-fine semantic reconstruction and spatial resolution recovery. The specific output dimensions of each decoder stage are as follows: Up1 output (512 channels, 64 × 64), Up2 output (256 channels, 128 × 128), Up3 (128 channels, 256 × 256) and Up4 output (64 channels, 512 × 512). Between the decoder output and the segmentation layer, the Edge-Guided Refinement Attention (EGRA) module is embedded. Through explicit edge extraction and dual-attention modulation, EGRA enhances high-frequency detail information in weak boundary and adherent regions, achieving synergistic enhancement of edge and semantic features. This effectively mitigates boundary blurring and adjacent tissue adhesion misclassification, ultimately producing a high-precision hippocampal segmentation result with the same spatial resolution as the input image.

#### 2.2.2 Salient Feature Enhancer module (SFE)

**Figure 2:**
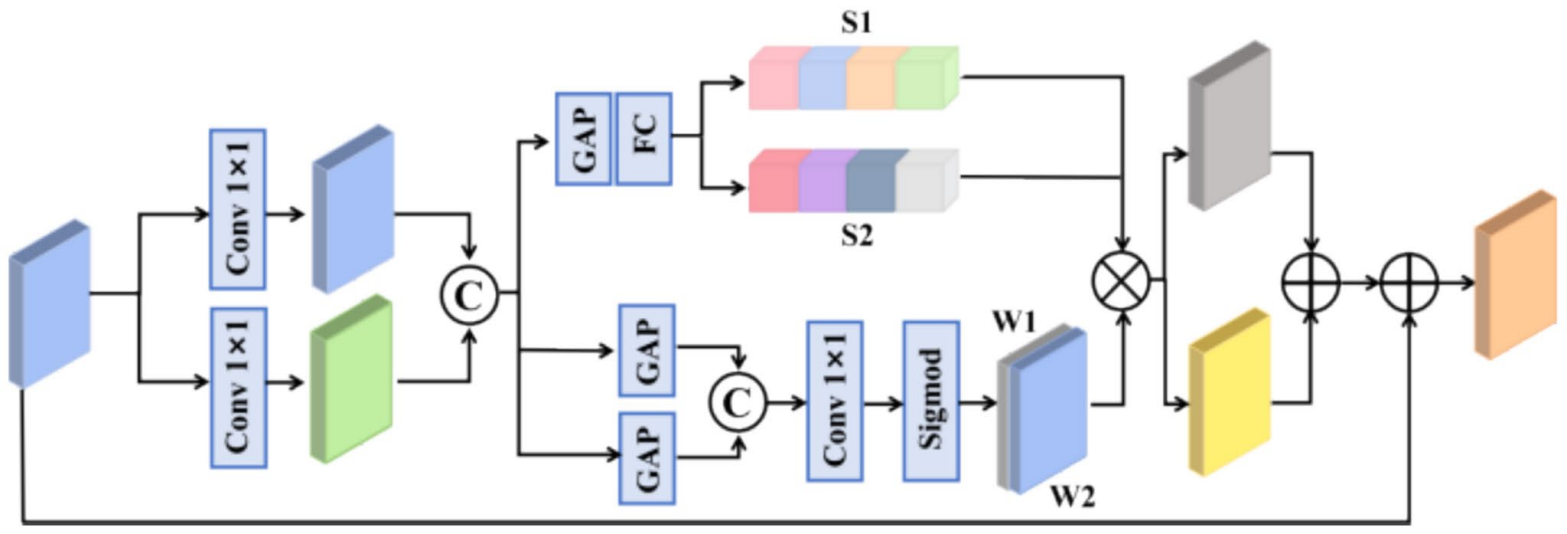
Salient Feature Enhancer (SFE).

To address the challenges in hippocampal segmentation—namely, the susceptibility of small-target features to being overwhelmed by brain tissue backgrounds, insufficient weak-boundary representation capability, and poor multi-scale adaptability—this paper designs the SFE, the structure is illustrated in Figure 2. This module integrates a dual-attention mechanism of channel and spatial attention. Without incurring significant computational overhead, it performs adaptive feature calibration and target enhancement. The SFE module can be embedded into each level of the encoder to achieve full-scale feature optimization, thereby providing highly discriminative feature support for subsequent multi-scale fusion and global modeling.

Given an input feature $F_{\text{in}}\in R^{C\times H\times W}$ , SFE employs two parallel 1 × 1 convolutional branches to extract detailed ( $F_{1}$ ) and semantic ( $F_{2}$ ) representations. The concatenated dual-branch features are processed by global average pooling to aggregate global channel statistics, followed by a squeeze-activation-expand structure to generate channel attention weights, which are then split into $S_{1}\;$ and $S_{2}$ for channel-wise modulation of the two branches, respectively. For spatial attention, global average and max pooling are applied, concatenated, and passed through a 7 × 1 convolution and Sigmoid to produce spatial masks $W_{1}$ and $W_{2}$ , adaptively enhancing hippocampal regions while suppressing background interference. The module simultaneously applies both channel and spatial weights to the dual-branch features, accomplishing dual-dimensional adaptive enhancement. This process is formulated as Equations 1,2:

$${F_{1}}^{'}=F_{1}⊗S_{1}⊗W_{1}$$ (1)

$${F_{2}}^{'}=F_{2}⊗S_{2}⊗W_{2}$$ (2)

The two attention-enhanced branch features are first summed element-wisely and then processed via a convolution block to obtain an intermediate fused feature. A residual shortcut is subsequently adopted to retain the original input feature information, which yields the final calibrated feature as shown in Equations 3,4. The final output is then obtained by adding the original input feature, as shown in Equation 5:

$$F_{\mathrm{mid}}=\text{ConVBNReLU}(F_{1}^{'}+F_{2}^{'})$$ (3)

$$F_{\mathrm{att}}=x+F_{\mathrm{mid}}$$ (4)

$$F_{\text{out}}=F_{\text{in}}+F_{\mathrm{att}}$$ (5)

SFE employs dual-branch separated representation, dual-attention cooperative modulation, and residual enhancement. It suppresses redundant background features (e.g., ventricles, gray matter) via global statistical modeling, alleviating small-target submergence. Simultaneously, it enhances weak-boundary and fine-region responses from channel and spatial dimensions, improving adaptability to hippocampal scale and morphological variations. Using only 1 × 1 convolutions and lightweight pooling, SFE maintains low parameter and computational costs, enabling easy embedding into each encoder stage for progressive multi-scale feature refinement. This provides high-quality features for subsequent MCFRM cross-scale fusion, GCCA global modeling, and edge-precise segmentation.

#### 2.2.3 Global Channel Context Attention (GCCA) module

**Figure 3:**
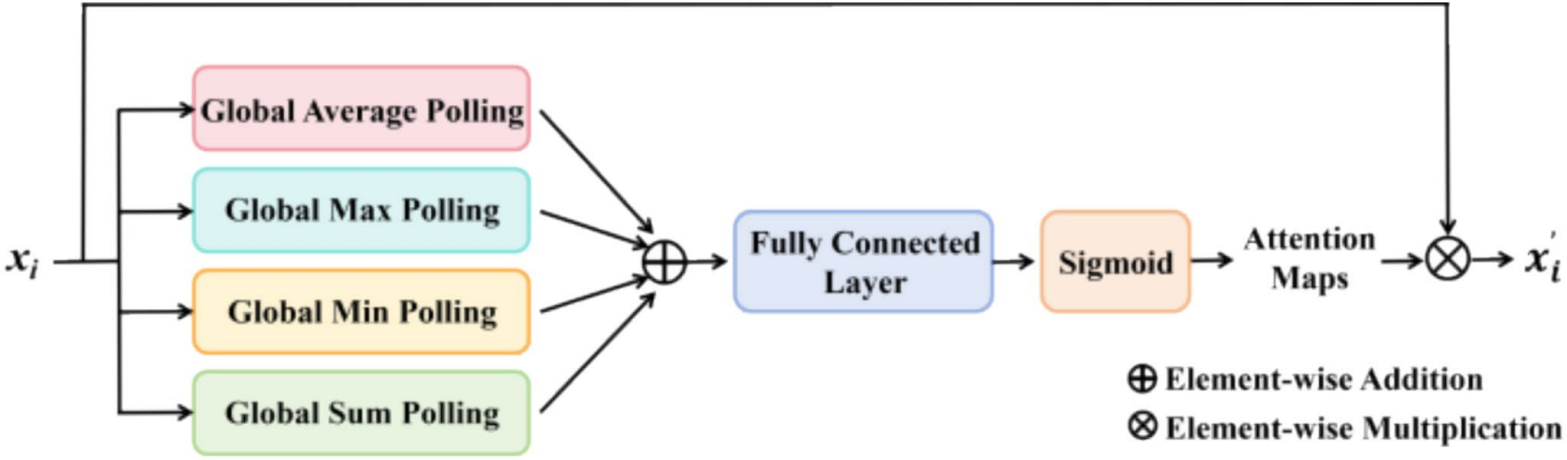
Global Channel Context Attention (GCCA).

The elongated and irregular spatial distribution of the hippocampus necessitates efficient global context modeling capabilities to mitigate the issues of missed segmentation and contour discontinuity. Traditional self-attention mechanisms, however, incur substantial computational overhead and are susceptible to noise interference, making it challenging to balance accuracy and efficiency. To address this, we propose the GCCA module, as illustrated in Figure 3. Deployed at the topmost encoder level, this module lightweight models global channel dependencies, providing stable high-level semantic guidance for the decoder.

The core of GCCA lies in multi-dimensional global statistical information aggregation and adaptive weighting. Given a feature map $x_{i}\in R^{B\times C\times H\times W}$ , a multi-branch global pooling operation is first applied, comprising Global Average Pooling (GAP), Global Maximum Pooling (GMP), Global Minimum Pooling (GMN), and Global Sum Pooling (GSP). These branches extract channel-wise features from the perspectives of mean, extreme values, lower bound, and global energy, thereby addressing the information loss issue inherent in single-pooling strategies. The features from all branches are then fused, and a lightweight fully connected layer is employed to learn nonlinear inter-channel dependencies, generating channel attention weights. Finally, the weights are multiplied element-wise with the original features to enhance critical channels and suppress ineffective ones. The core process is shown in Equations 6,7.

$$A=\sigma (\mathrm{FC}(\text{Aggregate}(\text{Pooling Branches}(x_{i}))))\;$$ (6)

$$x_{i}^{'}=x_{i}⊙A$$ (7)

Where Pooling Branches(·) denotes the multi-dimensional global pooling operation (GAP, GMP, GMN, GSP), Aggregate(·) is the multi-branch feature fusion function, and σ is the Sigmoid activation function producing attention weights within [0, 1].

GCCA achieves a balance between performance and efficiency through three key mechanisms. First, its core complexity is O(C) , significantly lower than that of traditional self-attention. Second, multi-dimensional information aggregation effectively filters out background noise and enhances the perception of irregular morphology and weak boundaries. Third, by operating on the highest-level features, it provides robust global guidance for the decoder. Specifically designed for the hippocampus, this module effectively captures global topological associations, alleviates the issues of missed segmentation and contour discontinuity, while maintaining controllable parameter overhead, thereby providing high-quality global feature support for subsequent semantic reconstruction.

#### 2.2.4 Multi-scale Context Fusion Refinement Module (MCFRM)

**Figure 4:**
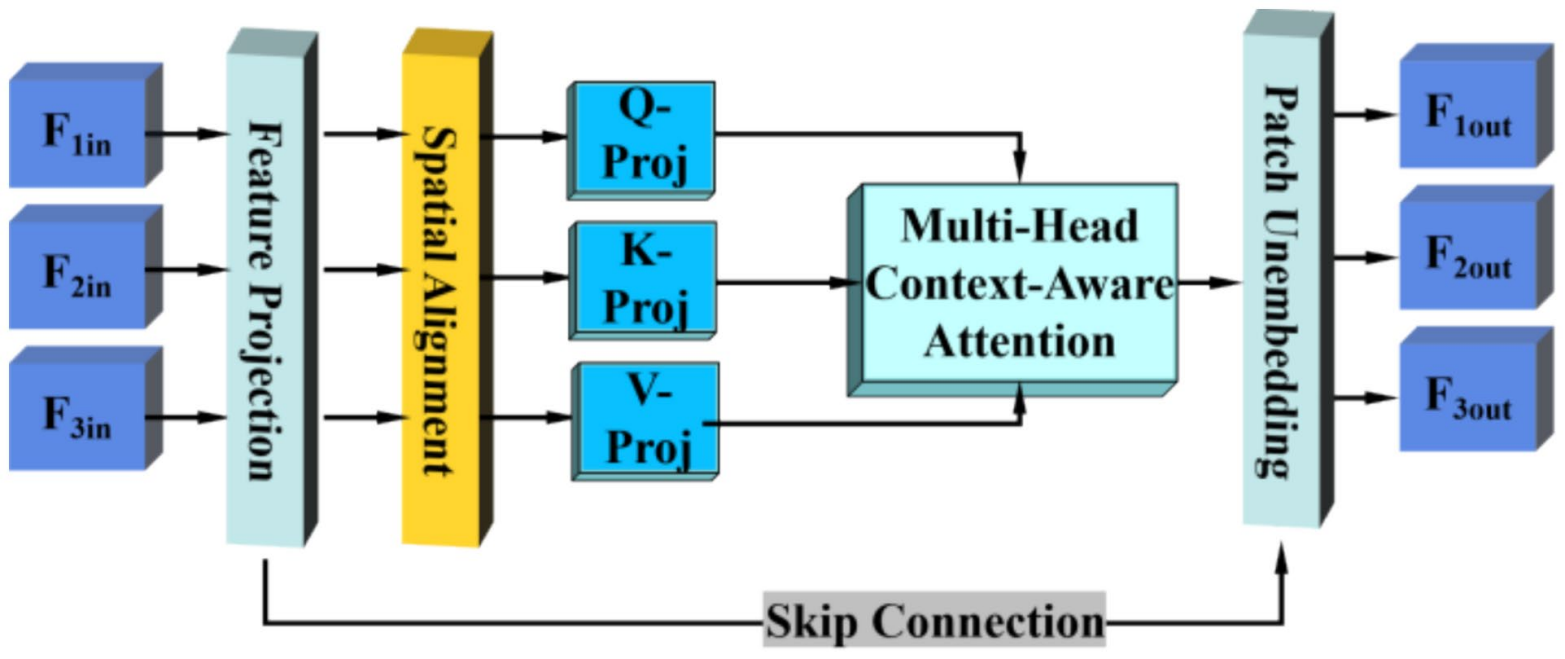
Multi-scale Context Fusion Refinement Module (MCFRM).

To overcome the issues of inconsistent channel dimensions and spatial misalignment of encoder multi-scale features, cross-level semantic discontinuity, low skip connection utilization, and insufficient long-range context modeling in hippocampal segmentation, this paper proposes a MCFRM (Figure 4). With a built-in Multi-scale Context-Aware Attention (MSCAA) submodule, MCFRM takes three-level encoder features as input and performs preprocessing alignment, attention-specific projection, context modeling, and residual refinement for end-to-end multi-scale feature enhancement. This provides reliable skip connection guidance to the decoder, significantly improving segmentation performance across hippocampi of varying scales and morphologies.

**Figure 5:**
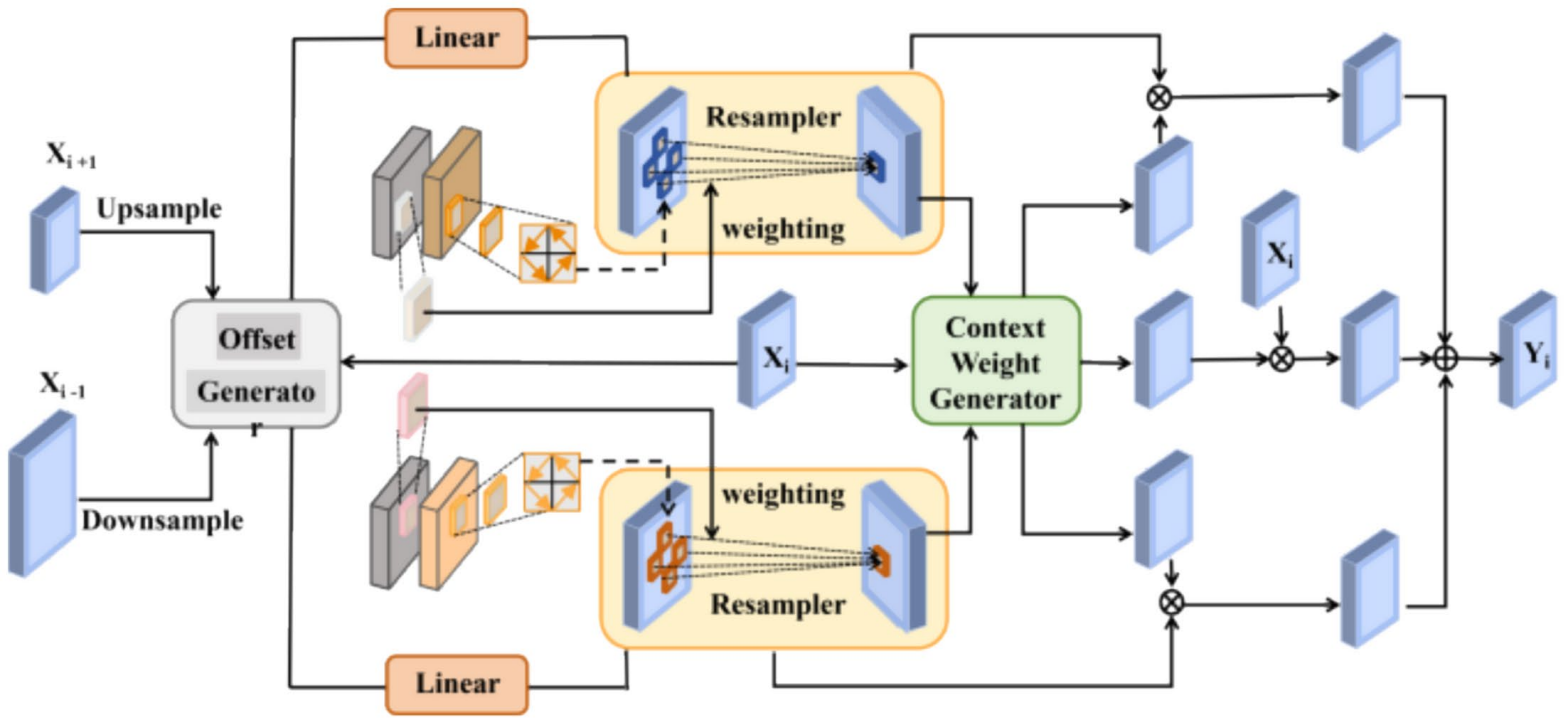
Multi-scale Context-Aware Attention (MSCAA).

The overall workflow of MCFRM is illustrated in Figure 5. First, a 1 × 1 convolutional projection maps the input features with varying channel dimensions to a unified intermediate channel dimension $C_{\mathrm{mid}}$ , eliminating fusion barriers caused by cross-level channel discrepancies. Subsequently, bilinear interpolation is applied to align spatial resolutions, yielding aligned features ${\tilde{F}}_{1},{\tilde{F}}_{2},{\tilde{F}}_{3}$ , which are then fused via element-wise averaging to obtain a globally fused feature $F_{\text{fuse}}$ , integrating complementary multi-level information and laying the foundation for subsequent attention modeling.

Beyond this basic fusion, MCFRM performs dedicated linear projections on the aligned multi-scale features ${\tilde{F}}_{1},{\tilde{F}}_{2},{\tilde{F}}_{3}$ to generate query ( Q ), key ( K ), and value ( V ) matrices for subsequent multi-head context attention computation. The multi-head scaled dot-product attention mechanism is then employed to capture long-range dependencies across multi-scale features, and the expression is shown in Equation 8:

$$\text{Attn}(Q,K,V)=\text{Softmax}(\frac{QK^{T}}{\sqrt{d_{k}}})V$$ (8)

Where $d_{k}$ is the dimension normalization scaling factor that prevents excessively large inner product values in high-dimensional features from causing Softmax gradient saturation. The resulting attention features, along with the original Q,K,V matrices, are passed to the built-in Multi-scale Context-Aware Attention (MSCAA) submodule (Figure 5). MSCAA first uses an offset generator to learn spatial offsets across feature levels, followed by a deformable resampler for fine-grained spatial alignment. A context weight generator then dynamically computes scale-wise contribution weights to adaptively rectify the globally fused feature, expressed as Equation 9:

$$F_{\text{context}}=W_{1}⊙{\tilde{F}}_{1}+W_{2}⊙{\tilde{F}}_{2}+W_{3}⊙{\tilde{F}}_{3}$$ (9)

Where ⊙ denotes element-wise multiplication. This dynamic weighting mechanism adaptively suppresses background redundancy and enhances hippocampal structural features, effectively mitigating cross-level feature fragmentation.

To prevent feature information degradation, the module employs a residual structure to fuse the attention-enhanced features with the original fused features. The enhanced features are then projected back to their original channel dimensions via a Patch Unembedding transposed convolution, followed by a second residual fusion through skip connections, ultimately producing decoder-adapted refined features $F_{1}^{\text{out}},F_{2}^{\text{out}},F_{3}^{\text{out}}$ .

Compared with single-scale attention and standard skip connections, MCFRM employs a lightweight closed-loop architecture to simultaneously resolve dimensional and spatial misalignments in multi-scale features. By integrating local details with global semantics via contextual attention, it substantially improves feature utilization and long-range modeling, providing the decoder with highly discriminative multi-scale features. This significantly enhances segmentation accuracy and robustness for hippocampi of varying scales and morphologies.

#### 2.2.5 Edge-Guided Refinement Attention (EGRA) module

**Figure 6:**
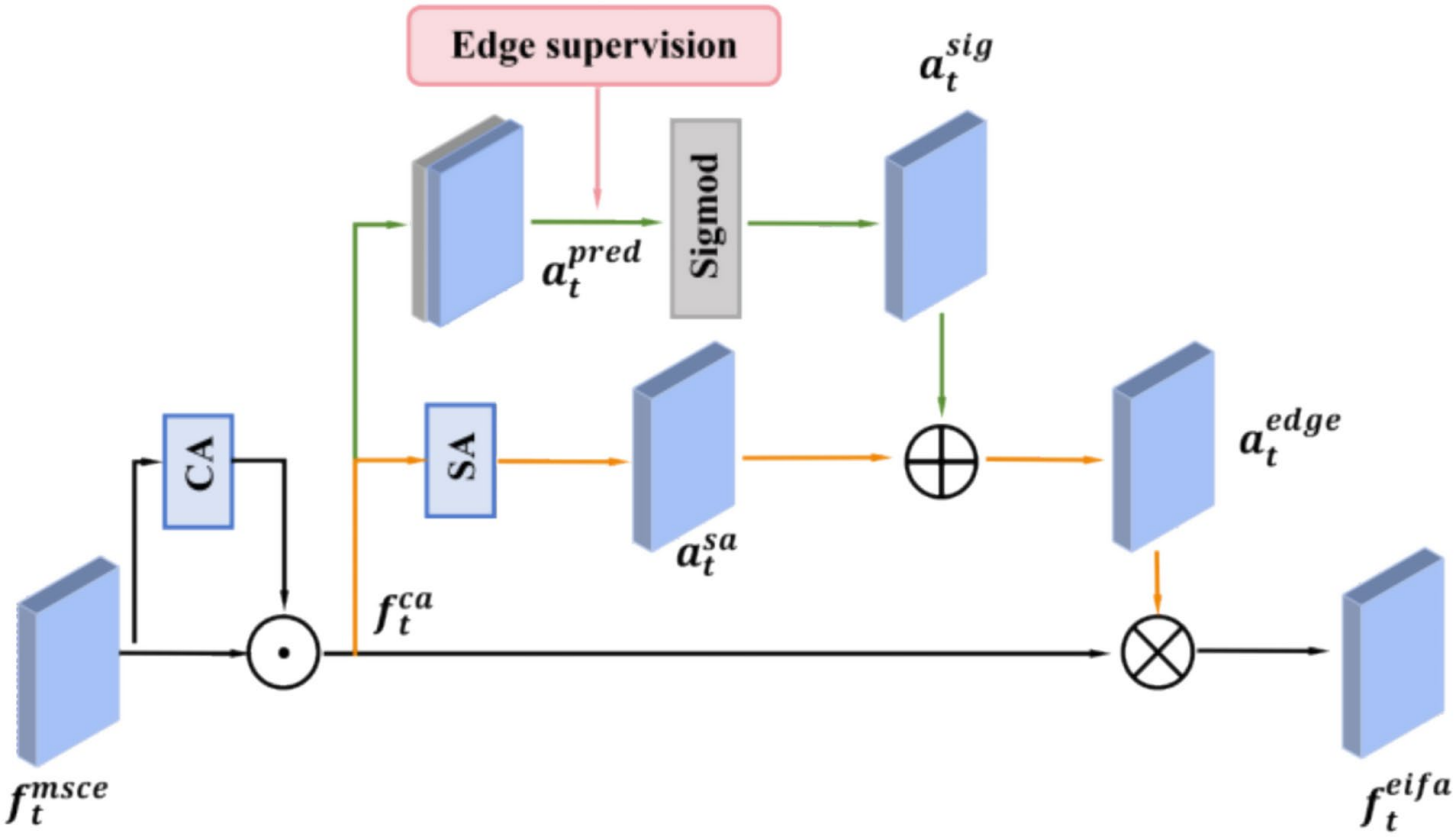
Edge-Guided Refinement Attention (EGRA).

To address the challenges of boundary blurring and adjacent tissue adhesion in hippocampal segmentation, which hinder precise contour extraction, this paper introduces an EGRA module at the decoder output stage, with its structure illustrated in Figure 6. This module embeds explicit edge supervision into the spatial attention mechanism, achieving synergistic enhancement of semantic and edge features, precisely strengthening weak boundary representations while suppressing spurious edge interference from the background.

EGRA takes the high-resolution feature from the final upsampling stage of the decoder as input. It first employs channel attention (CA) to adaptively enhance key semantic channels, producing a channel-enhanced feature $f_{t}^{\mathrm{ca}}$ . Subsequently, $f_{t}^{\mathrm{ca}}$ is split into two parallel branches: the edge branch predicts the target edge map $a_{t}^{\text{pred}}$ through two convolutional layers, with explicit supervision provided by edge labels extracted from the ground truth segmentation using the Sobel operator; the spatial branch generates a spatial saliency attention map $a_{t}^{\mathrm{sa}}$ via spatial attention (SA). The predicted edge map is normalized by Sigmoid and then element-wise added to the spatial attention map to produce the fused edge attention map $a_{t}^{\text{edge}}$ . Finally, the fused attention map is element-wise multiplied with the channel-enhanced feature, followed by convolutional refinement and a residual connection to complete feature optimization, outputting the edge-enhanced feature $f_{t}^{\text{eifa}}$ .

EGRA integrates channel-spatial attention coupling with explicit edge supervision to adaptively enhance hippocampal semantic channels and spatial contours while leveraging edge priors to suppress adherent tissue and background noise. This effectively refines weak boundaries and compensates for contour discontinuities, delivering highly discriminative edge-semantic features for final segmentation.

#### 2.2.6 Loss function

To address the challenges of extremely high background pixel proportion, sparse left and right hippocampal regions, and severe class imbalance in brain hippocampal segmentation, this paper adopts a weighted combination of class-weighted multi-class cross-entropy loss and multi-class Dice loss as the optimization objective for the network. This loss alleviates class imbalance while enhancing segmentation accuracy and contour continuity of the hippocampal regions. The expression is shown in Equation 10:

$$L_{\text{total}}=\lambda_{1}L_{\mathrm{wce}}+\lambda_{2}L_{\text{dice}}$$ (10)

Where $\lambda_{1}$ and $\lambda_{2}$ are the balance coefficients for the weighted cross-entropy loss and the Dice loss, respectively. Given the severe class imbalance and sparse foreground of hippocampal segmentation, we set a larger weight for Dice loss to focus on foreground structure optimization. Referring to common empirical settings in medical image segmentation, we empirically set λ_1_ = 0.4 and λ_2_ = 0.6. This configuration ensures stable training and satisfactory segmentation performance.

The dataset in this paper adopts a three-class annotation scheme, with background labeled as 0, right hippocampus as 1, and left hippocampus as 2, representing a typical multi-class small-target segmentation scenario. Standard cross-entropy loss tends to be dominated by the large number of background pixels, resulting in insufficient learning of the sparse hippocampal regions. To address this issue, we introduce a class-weighted multi-class cross-entropy loss, which assigns higher loss weights to the hippocampal classes to alleviate sample imbalance between foreground and background as well as across different classes, and the expression is shown in Equation 11:

$$L_{\mathrm{wce}}=-\frac{1}{N}\sum_{i=1}^{N}\sum_{c=0}^{2}\omega_{c}\cdot m_{i,c}\log p_{i,c}$$ (11)

Where N is the total number of pixels in the image; c denotes the class index ( c=0 for background, c=1 for right hippocampus, c=2 for left hippocampus); $m_{i,c}$ is the one-hot label of pixel i for class c ; $p_{i,c}$ is the class probability output by the model after Softmax activation; and $\omega_{c}$ is the class weight. Based on the pixel ratio between background and hippocampus in the dataset, the class weights are set as $\omega_{0}=0.4$ (background), $\omega_{1}=2.5$ (right hippocampus), and $\omega_{2}=2.5$ (left hippocampus), to enhance the model’s attention to the sparse hippocampal regions.

To further optimize the regional overlap accuracy and weak boundary continuity of the left and right hippocampi, the multi-class Dice loss is introduced to directly optimize region-wise similarity across classes, compensating for the limitation of cross-entropy loss that operates only at the pixel level, the Equation 12 as follows:

$$L_{\text{dice}}=1-\frac{1}{C}\sum_{c=0}^{2}\frac{2\sum_{i=1}^{N}p_{i,c}m_{i,c}+\epsilon }{\sum_{i=1}^{N}p_{i,c}+\sum_{i=1}^{N}m_{i,c}+\epsilon }$$ (12)

Where $\in =10^{-6}$ is a smoothing factor to prevent division by zero, and C=3 is the total number of classes. The multi-class Dice loss is insensitive to class distribution and effectively alleviates the sample imbalance problem, improving the segmentation continuity of the elongated hippocampus with weak boundaries.

During training, the balance coefficients are set to $\lambda_{1}=0.6$ and $\lambda_{2}=0.5$ . This combined loss integrates pixel-level classification constraints with region-level overlap optimization. It ensures training convergence stability through the weighted cross-entropy loss while leveraging the Dice loss to focus on precise segmentation of the left and right hippocampi. This design effectively addresses the challenges inherent in brain hippocampal segmentation, including class imbalance, boundary ambiguity, and the elongated and irregular morphology of the target, providing stable and robust optimization supervision for the network.

#### 2.2.7 Datasets and evaluation metrics

**Figure 7:**
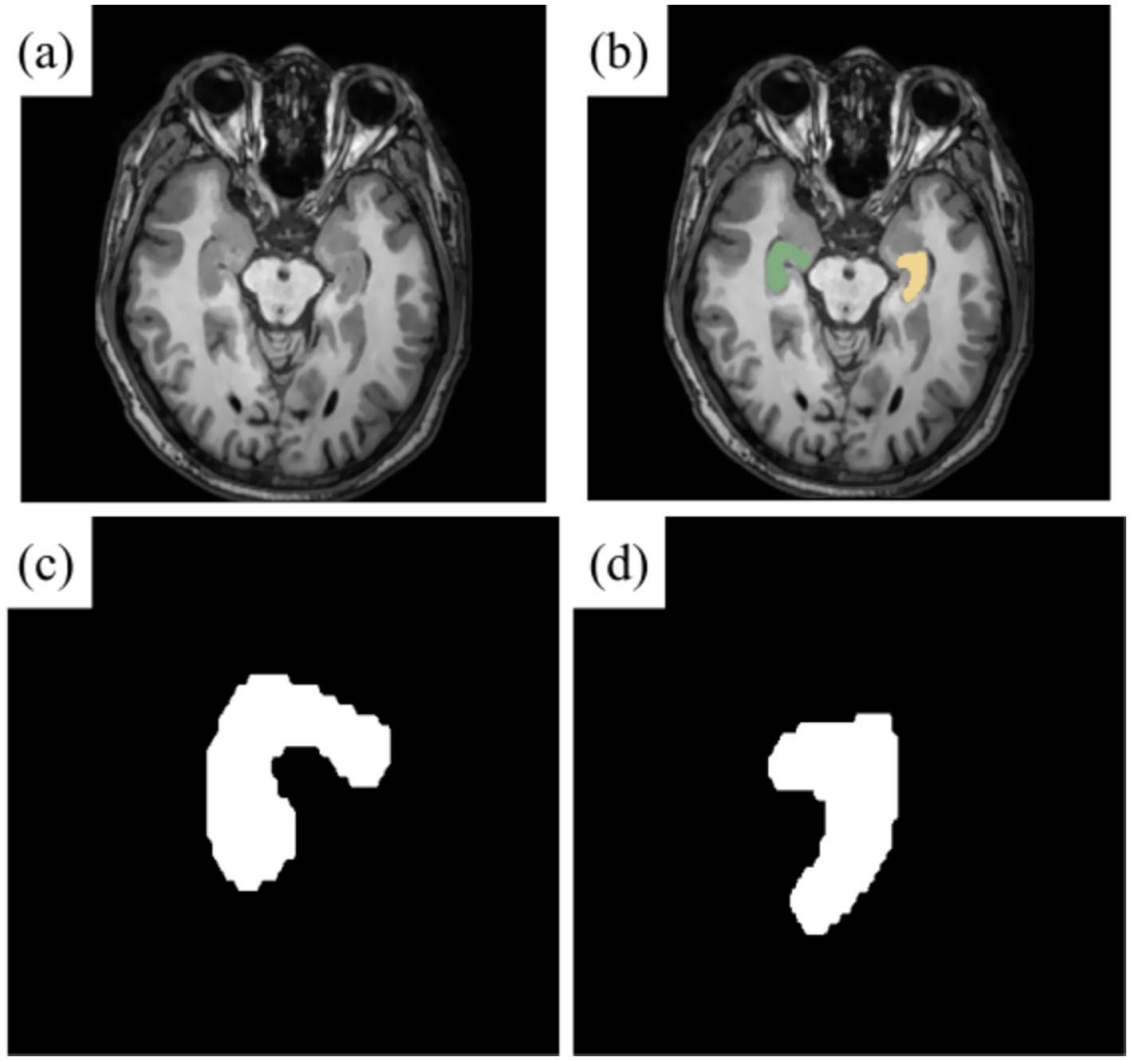
Example of hippocampus segmentation. **(a)** Original MR image, **(b)** Label sample, **(c)** right hippocampus segmentation map, **(d)** left hippocampus segmentation map.

We employ a self-constructed brain hippocampal segmentation dataset, comprising a total of 225 clinical brain MR scans, which were manually and meticulously annotated by radiologists. The dataset label definition rules are as follows: background pixels are labeled as 0, right hippocampus pixels as 1, and left hippocampus pixels as 2. Representative data samples are shown in Figure 7. In the experiments, all cases were randomly divided into training, validation, and test sets at a ratio of 8:1:1 for model training, parameter optimization, and final performance evaluation, respectively. All MRI images underwent unified spatial normalization and intensity standardization preprocessing to eliminate interference from different scanning equipment, magnetic field inhomogeneity, and individual variations, ensuring data distribution consistency. All slices were resampled to a resolution of 512 × 512.

The experimental hardware platform was configured with an Intel(R) Core (TM) i9-13900KF @ 3.00GHz processor, an NVIDIA GeForce RTX 4090 GPU, and 64 GB of RAM. All experiments were conducted using the PyTorch 2.1 deep learning framework and Python 3.9 environment. Input images were uniformly normalized to a size of 512 × 512 × 1, with a batch size of 8. The Adam algorithm was adopted as the optimizer, with an initial learning rate of 1e-4 and a weight decay coefficient of 1e-5. The learning rate decay strategy employed Cosine Annealing, enabling smooth and dynamic reduction of the learning rate during training to enhance convergence stability. The model was trained for a total of 200 epochs. Gradient clipping was applied to prevent gradient explosion, and an early stopping strategy was adopted to avoid overfitting, ensuring good generalization ability of the model in the small-target hippocampal segmentation task.

To comprehensively and quantitatively evaluate the segmentation accuracy and contour fitting performance of the proposed model for the left and right hippocampus, four mainstream metrics in the field of medical image segmentation are adopted: Dice Similarity Coefficient (DSC), Intersection over Union (IoU), 95% Hausdorff Distance (HD95), and Average Symmetric Surface Distance (ASD). Among these, Dice and IoU are used to measure the overall overlap between the predicted region and the ground truth region, while HD95 and ASD focus on quantifying the matching error of segmentation boundaries, making them particularly suitable for evaluating fine structures such as the hippocampus and their boundary precision. Let X denote the predicted segmentation region and Y denote the manually annotated ground truth region. The definitions and calculation formulas of the specific metrics are shown in Equations 13,14.

$$\text{Dice}=\frac{2\times |X\cap Y|}{|X|+|Y|}$$ (13)

$$\mathrm{IOU}=\frac{|X\cap Y|}{|X\cup Y|}$$ (14)

Dice and IoU measures the overall overlap between the predicted region and the standard label region. The higher the value of Dice and IoU, the closer the segmentation result of the algorithm is to the standard label, and the better the segmentation performance of the algorithm.

The 95% Hausdorff Distance (HD95) computes the 95th percentile of the voxel-wise distances between the predicted segmentation contour and the ground truth contour, mitigating the influence of extreme outliers. It quantitatively describes the maximum boundary deviation, with units in voxels. A smaller HD95 value indicates more precise boundary fitting. The expression is shown in Equation 15:

$$\begin{array}{l} \mathrm{HD}95=95\mathrm{th}\;\text{percentile}\\ \;\{\max_{x\in \partial X}\min_{y\in \partial Y}d(x,y),,\max_{y\in \partial Y}\min_{x\in \partial X}d(x,,y)\} \end{array}$$ (15)

$$\begin{array}{l} \mathrm{ASD}=\frac{1}{S(X)+S(Y)}\\ \times (\sum_{S_{X}\in S(X)}d(S_{X'}S(Y))+\sum_{S_{Y}\in S(Y)}d(S_{Y'}S(X))) \end{array}$$ (16)

The ASD computes the average of bidirectional surface distances between the predicted contour and the ground truth contour, providing an overall characterization of the average deviation between the segmentation surface and the standard contour. A smaller ASD value indicates better overall surface consistency, and the expression is shown in Equation 16. Here, S(X) and S(Y) denote the surface point sets of the predicted and ground truth regions, respectively, and d(·,·) represents the Euclidean distance. An ASD value closer to 0 indicates higher adherence of the segmentation boundary to the ground truth boundary, as well as smoother and more precise boundary delineation.

### 2.3 Statistical analysis

The normality of the data distribution was assessed using the Shapiro–Wilk test. To compare demographic characteristics and hippocampal volumes between HCs and MCI group, independent-samples *t* test or Mann–Whitney *U* test was performed, as appropriate. Receiver operating characteristic (ROC) analysis with the area under the curve (AUC) was conducted to identify the differential diagnostic performance of hippocampal volumes in distinguishing between HCs and MCI. All statistical analyses were conducted using SPSS version 26.0, MedCalc version 15.2.0, and GraphPad Prism version 8.2.1. A *p*-value <0.05 was considered statistically significant.

## 3 Results

### 3.1 Ablation experiment

**Figure 8:**
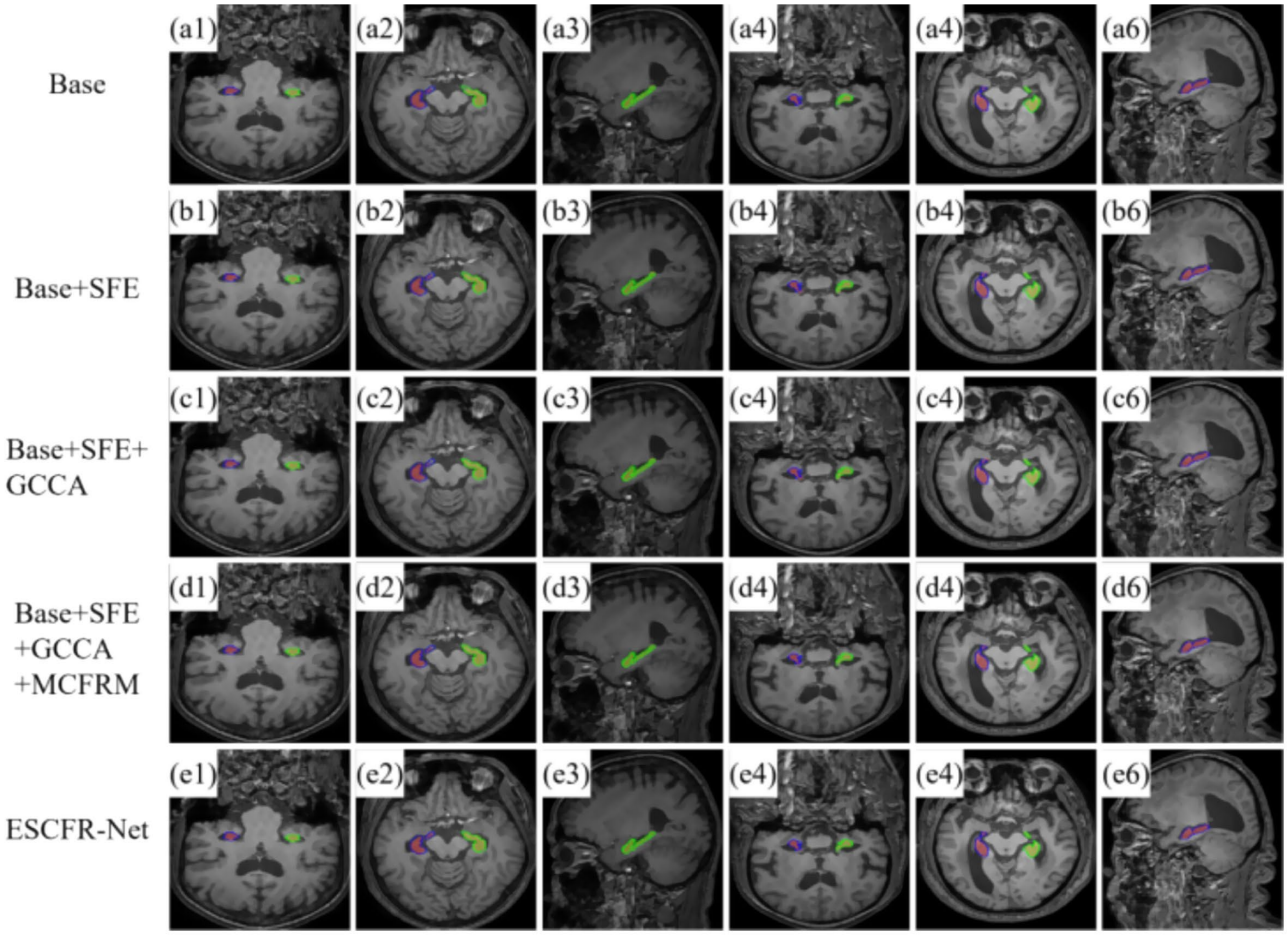
Comparison of ablation study results. **(a)** Base, **(b)** Base+SFE, **(c)** Base+SFE+GCCA, **(d)** Base+SFE+GCCA+MCFRM, **(e)** ESCFR-Net.

To quantitatively validate the individual contributions and synergistic gains of each core component of the proposed ESCFR-Net, and to reveal the improvements of each module in enhancing small-target weak features, global context modeling, multi-scale feature fusion, and edge contour refinement for brain hippocampal segmentation, we adopt the standard U-Net as the baseline network (Base). SFE, GCCA, MCFRM, and EGRA are progressively embedded to construct a series of incremental ablation models. All models are trained and evaluated on the self-constructed brain hippocampal dataset under identical experimental conditions, training hyperparameters, and data partitioning strategies. Quantitative evaluation is performed using four metrics: Dice, IoU, HD95, and ASD. Five comparative models are established: Base, Base+SFE (B+S), Base+SFE+GCCA (B+S+G), Base+SFE+GCCA+MCFRM (B+S+G+M), and Base+SFE+GCCA+MCFRM+EGRA (ESCFR-Net, B+S+G+M+E). The quantitative performance and segmentation visualization results of the above models are presented in Table 1 and Figure 8, respectively.

**Table 1:** Ablation study results of the proposed models.

Model	Dice	IoU	HD95 (mm)	ASD (mm)
Base	0.8295	0.7095	5.7359	2.4217
B+S	0.8369	0.7354	4.7602	1.7517
B+S+G	0.8720	0.7827	3.3074	0.8368
B+S+G+M	0.8873	0.8317	2.1546	0.6732
ESCFR-Net	0.9004	0.8466	1.5895	0.4625

Quantitative results in Table 1 show that compared to the Base network, SFE improves Dice from 0.8295 to 0.8369 and IoU from 0.7095 to 0.7354, with reduced HD95/ASD, indicating effective background suppression and weak feature enhancement. Adding GCCA further improves HD95 from 4.7602 to 3.3074, enhancing contour continuity and validating its lightweight global channel modeling for capturing topological associations of the elongated hippocampus. Incorporating MCFRM yields substantial improvements across all metrics (IoU: 0.8317, HD95: 2.1546), demonstrating its cross-scale feature alignment and fusion capabilities. ESCFR-Net with EGRA achieves optimal results (Dice: 0.9004, IoU: 0.8466, HD95: 1.5895, ASD: 0.4625), proving EGRA’s effectiveness in refining weak boundaries and adherent regions via edge supervision and attention.

Visual results in Figure 8 show progressive improvement in contour delineation and boundary continuity with each added module. Base network suffers from background noise and under-segmentation; Base+SFE shows improved overlap but over-segmentation due to limited global context; adding GCCA and MCFRM substantially reduces HD95/ASD, yielding better morphological consistency; ESCFR-Net achieves fine-grained weak-boundary segmentation while preserving topological integrity.

The ablation study confirms that all four modules provide positive, complementary, and synergistic gains, with progressive stacking continuously improving segmentation overlap, global topological modeling, and boundary fitting. These results fully validate the rationality and necessity of each module design and the effectiveness of ESCFR-Net for high-precision automatic hippocampal segmentation.

### 3.2 Comparison with medical image segmentation methods

**Figure 9:**
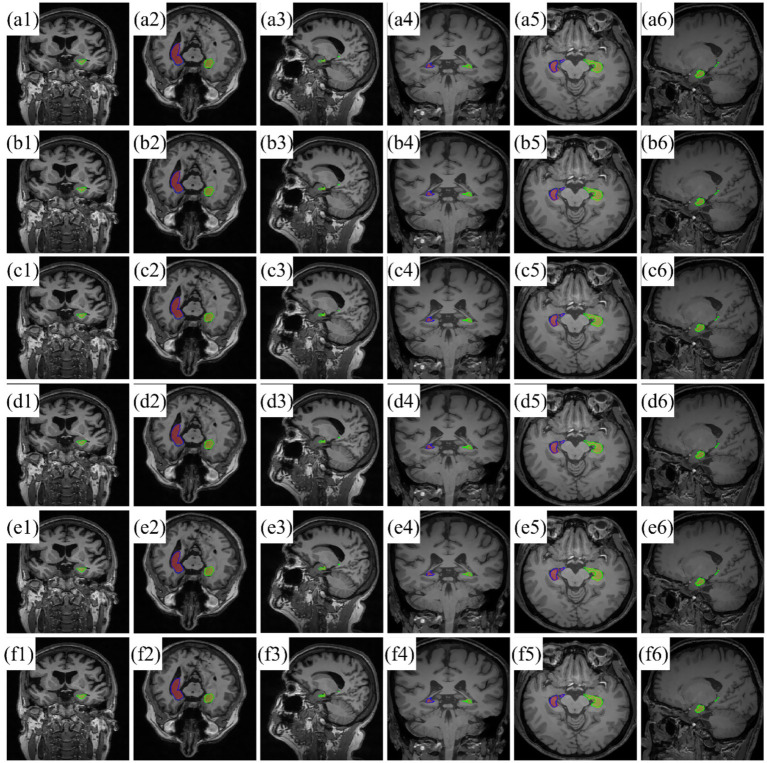
Comparison of hippocampal segmentation results using different networks. **(a)** SwinUNETR, **(b)** PMFS-Net, **(c)** Light3DHS, **(d)** DPNU-Net, **(e)** FED-UNet++, **(f)** ESCFR-Net.

To validate the effectiveness of the proposed ESCFR-Net in the task of brain hippocampal segmentation, we conducted comparative experiments against five mainstream medical image segmentation algorithms SwinUNETR (24), PMFS-Net (20), Light3DHS (19), DPNU-Net (23), and FED-UNet++ (22) on our self-constructed brain hippocampal dataset. The quantitative segmentation results of each model are presented in Table 2, and the visual comparison results are shown in Figure 9.

**Table 2:** Comparison of ESCFR-Net with other networks.

Method	Dice	IoU	HD95 (mm)	ASD (mm)
SwinUNETR	0.8460	0.7438	4.1165	1.1267
PMFS-Net	0.8706	0.7703	3.7867	0.8158
Light3DHS	0.8890	0.8099	2.7103	0.6973
DPNU-Net	0.8918	0.8361	1.9441	0.5717
FED-UNet++	0.8992	0.84562	1.6207	0.4630
ESCFR-Net	0.9004	0.8466	1.5895	0.4625

From the quantitative comparison results presented in Table 2, it can be observed that ESCFR-Net achieves the best performance across all metrics. Compared with other algorithms, ESCFR-Net attains a Dice coefficient of 0.9004 and an IoU of 0.8466, significantly outperforming SwinUNETR (Dice = 0.8460, IoU = 0.7438), PMFS-Net (Dice = 0.8706, IoU = 0.7703), Light3DHS (Dice = 0.8890, IoU = 0.8099), DPNU-Net (Dice = 0.8918, IoU = 0.8361), and FED-UNet++ (Dice = 0.8992, IoU = 0.8456). This demonstrates that the proposed model achieves higher overall segmentation overlap with the hippocampal region and better alignment with the ground truth. In terms of boundary contour metrics, ESCFR-Net achieves an HD95 of 1.5895 mm and an ASD of 0.4625 mm, both being the lowest values among all compared models. This indicates that the model achieves higher precision in delineating weak boundaries, adherent regions, and the elongated irregular structure of the hippocampus, effectively alleviating issues such as contour discontinuities, under-segmentation, and over-segmentation at the boundaries.

From the visualization results shown in Figure 9, it can be observed that Swin-UNet is susceptible to background noise, resulting in poor segmentation of edges and fine structures. PMFS-Net and Light3DHS exhibit limited capability in handling weak boundaries and adherent regions, with visible jagged boundaries and under-segmentation issues along the contours. DPNU-Net and FED-UNet++ achieve improved boundary precision, yet their global topological modeling remains deficient. In contrast, ESCFR-Net effectively balances global context modeling and local detail delineation. While preserving the topological integrity of the elongated hippocampal structure, it achieves fine-grained segmentation of weak boundaries and adherent regions, with segmentation contours that closely match the morphology of the ground truth hippocampus. These results validate its superiority in the task of high-precision brain hippocampal segmentation.

From both quantitative and qualitative results, it can be concluded that ESCFR-Net, through the integration of salient feature enhancement, global context modeling, multi-scale feature fusion, and edge-guided refinement modules, effectively overcomes the challenges faced by conventional networks in hippocampal segmentation, including significant background interference, insufficient feature utilization, and poor contour fitting. It outperforms existing mainstream algorithms in both overall segmentation accuracy and boundary delineation capability, demonstrating stronger potential for practical clinical application.

### 3.3 Participant characteristics of healthy controls and patients with MCI

Table 3 shows the demographic characteristics of the participants. A total of 91 participants were enrolled in the MCI group, and 91 were assigned to the HCs. No statistically significant differences were found between the two groups in sex, age, BMI, or years of education (all *p* > 0.05). Compared with the HCs, the MCI group had significantly lower MMSE scores (*p* < 0.001).

**Table 3:** Demographics of healthy controls and patients with MCI.

Variables	HC (*n* = 91)	MCI (*n* = 91)	*p*
Sex (male/female)	48/43	40/51	0.394
Age (years)	65.8 ± 7.2	68.7 ± 6.8	0.938
BMI	25.77 ± 4.97	25.53 ± 3.91	0.387
Education (years)	9.4 ± 4.6	9.6 ± 5.2	0.177
MMSE (score)	28.7 ± 1.3	22.8 ± 3.4	<0.001

HC, healthy control; MCI, mild cognitive impairment; MMSE, mini-mental state examination.

### 3.4 Comparison of hippocampal volumes between HC and MCI group

**Figure 10:**
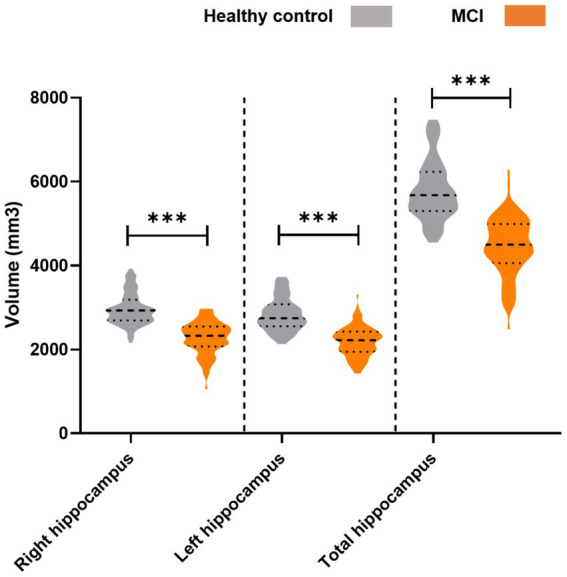
Violin plots of right, left, and total hippocampal volumes between healthy controls and patients with mild cognitive impairment (MCI). Statistically significant, ^*^p < 0.05, ^**^p < 0.01, ^***^p < 0.001.

Table 4 presents right, left, and total hippocampal volumes between HCs and MCI group. The mean right, left, and total hippocampal volumes for the HC group were 2,957.8 mm^3^, 2,834.2 mm^3^, and 5,792.0 mm^3^, respectively. For the cognitive impairment group, the corresponding values were 2,274.1 mm^3^, 2,184.6 mm^3^, and 4,458.7 mm^3^, separately. Compared with HCs, the MCI group had significantly lower volumes in all three measures (all *p* < 0.001) (Figure 10).

**Table 4:** Comparison of right, left, and total hippocampal volumes between healthy controls and patients with MCI.

Variables	HC	MCI	*p*
Right hippocampal volume (mm^3^)	2,957.8 ± 379.4	2,274.1 ± 387.5	<0.001
Left hippocampal volume (mm^3^)	2,834.2 ± 395.4	2,184.6 ± 359.0	<0.001
Total hippocampal volume (mm^3^)	5,792.0 ± 720.1	4,458.7 ± 694.4	<0.001

Data represent mean ± SD. HC, healthy control; MCI, mild cognitive impairment.

### 3.5 Differential diagnostic performance of hippocampal volumes in distinguishing between healthy controls and patients with MCI

**Figure 11:**
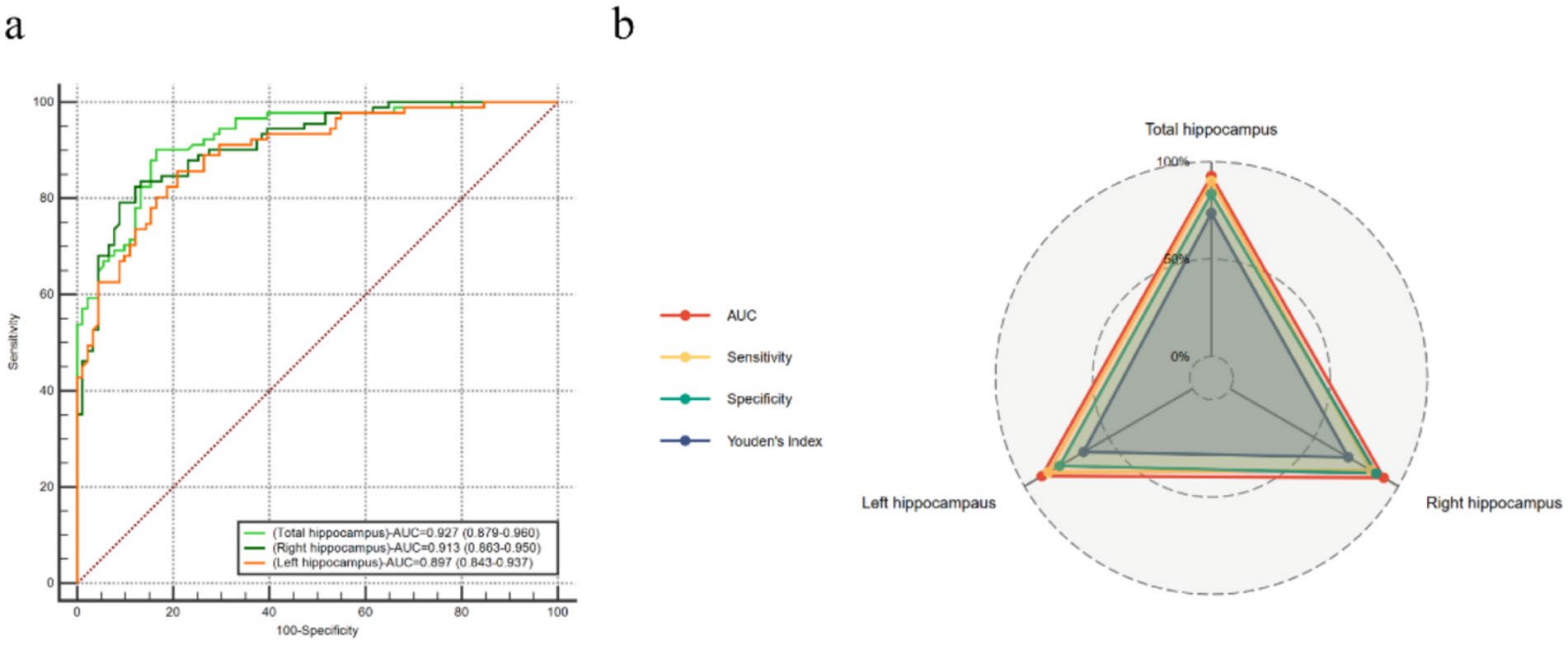
Receiver operating characteristic (ROC) curves **(a)** and Radar chart **(b)** based on right, left, and total hippocampal volumes for differentiating between patients with mild cognitive impairment (MCI) and healthy controls.

Figure 11 illustrates the differential diagnostic performance of right, left, and total hippocampal volumes in distinguishing between HC and MCI patients. The AUC values for the right, left, and total hippocampal volumes were 0.913, 0.897, and 0.927, respectively. The corresponding sensitivities were 83.52, 85.71, and 90.11%, respectively, and the specificities were 86.81, 79.12, and 83.52%, respectively. Overall, the right, left, and total hippocampal volumes demonstrated effective differential diagnostic performance.

## 4 Discussion

Hippocampal segmentation in medical images holds significant research value for neuroimaging quantitative analysis, auxiliary diagnosis of brain diseases, and clinical evaluation, as it can assist physicians in achieving precise morphological assessment of the hippocampus and individualized diagnostic decision-making (32, 33). However, due to challenges such as the elongated morphology, large scale variations, blurred boundaries, adjacent tissue adhesion, and susceptibility of small-target features to being overwhelmed, achieving high-precision automatic segmentation remains difficult. To improve segmentation accuracy, we propose the ESCFR-Net based on a U-shaped architecture, incorporating four core modules—SFE, GCCA, MCFRM, and EGRA—to accomplish small-target feature enhancement, global context modeling, multi-scale feature fusion, and weak boundary contour refinement, respectively. A compound loss function is further adopted to alleviate sample class imbalance. These designs enrich effective feature representations while suppressing irrelevant background interference, thereby improving network segmentation performance and robustness. Experimental results demonstrate that our method achieves a Dice coefficient of 0.9004 and an IoU of 0.8466, outperforming several existing mainstream algorithms in the task of automatic hippocampal segmentation.

Our findings demonstrate a significant association between reduced hippocampal volumes and cognitive impairment, consistent with previous MRI-based studies (34–37). The hippocampus plays a critical role in memory consolidation and spatial navigation, and its structural integrity is known to be vulnerable to neurodegenerative processes (38, 39). In this study, patients with MCI exhibited significantly smaller right, left, and total hippocampal volumes than healthy controls, suggesting that bilateral hippocampal atrophy is a hallmark of cognitive decline. Furthermore, the diagnostic performance of hippocampal volumetry, with AUC values exceeding 0.89 for all three measures, supports its utility as a non-invasive imaging biomarker for differentiating cognitive impairment from normal aging. Notably, total hippocampal volume yielded the highest AUC (0.927) and sensitivity (90.11%), implying that combining information from both cerebral hemispheres may provide the most robust diagnostic power. These results align with previous studies demonstrating that hippocampal atrophy progresses with disease severity (40–42), which reinforces the central role of hippocampal degeneration in cognitive impairment and highlight the potential of automated volumetry in clinical assessment.

The proposed method still has certain limitations. First, brain MRI images are characterized by low grayscale contrast and complex background tissues, leaving room for further improvement in the model’s accuracy in segmenting subtle hippocampal regions. Subsequent work could introduce post-processing strategies based on the coarse segmentation to obtain finer segmentation contours. Second, the dataset used in this study comes from a single center. In future work, multi-center clinical data will be incorporated to conduct cross-device and cross-scenario validation, thereby further enhancing the model’s generalization capability. Finally, this study confirms a cross-sectional association between hippocampal volume and mild cognitive impairment, the practical clinical value of the model in longitudinal follow-up, early warning, and disease progression prediction still needs to be further substantiated by large-scale, prospective cohort studies.

## 5 Conclusion

We propose an Edge-aware Salient Context Fusion Refinement Network (ESCFR-Net) for hippocampus segmentation on MR image in this paper. By organically integrating four core modules, namely salient feature enhancement, global context modeling, multi-scale feature fusion, and edge-guided refinement, ESCFR-Net obtain a more accurate feature representation. These modules cooperate complementarily to refine feature representation and acquire accurate feature description. Further clinical analysis reveals that hippocampal volume atrophy is closely associated with mild cognitive decline, and bilateral hippocampal volume measurements exhibit favorable diagnostic discrimination efficacy. This study offers a robust and reliable technical solution for automated and high-precision hippocampal segmentation in brain MRI, paving the way for the clinical application of imaging biomarkers for the monitoring and assessment of Alzheimer’s disease and related neurodegenerative disorders.

## Data Availability

The raw data supporting the conclusions of this article will be made available by the authors, without undue reservation.
